# Central Sensitization in Neurological, Psychiatric, and Pain Disorders: A Multicenter Case-Controlled Study

**DOI:** 10.1155/2021/6656917

**Published:** 2021-02-15

**Authors:** Keisuke Suzuki, Yasuo Haruyama, Gen Kobashi, Toshimi Sairenchi, Koji Uchiyama, Shigeki Yamaguchi, Koichi Hirata

**Affiliations:** ^1^Department of Neurology, Dokkyo Medical University, Mibu, Japan; ^2^Department of Public Health, Dokkyo Medical University School of Medicine, Mibu, Japan; ^3^Laboratory of International Environmental Health, Center for International Cooperation, Dokkyo Medical University, Mibu, Japan; ^4^Department of Anesthesiology, Dokkyo Medical University School of Medicine, Mibu, Japan

## Abstract

**Background:**

The role of central sensitization in refractory pain-related diseases has not yet been clarified.

**Methods:**

We performed a multicenter case-controlled study including 551 patients with various neurological, psychological, and pain disorders and 5,188 healthy controls to investigate the impact of central sensitization in these patients. Symptoms related to central sensitization syndrome (CSS) were assessed by the Central Sensitization Inventory (CSI) parts A and B. Patients were categorized into 5 groups based on CSI-A scores from subclinical to extreme. The Brief Pain Inventory (BPI), addressing pain severity and pain interference with daily activities, and the Patient Health Questionnaire (PHQ)-9, assessing depressive symptoms, were also administered.

**Results:**

CSI-A scores and CSI-B disease numbers were significantly greater in patients than in controls (*p* < 0.001). Medium effect sizes (*r* = 0.37) for CSI-A scores and large effect sizes (*r* = 0.64) for CSI-B disease numbers were found between patients and control groups. Compared with the CSI-A subclinical group, the CSI-A mild, moderate, severe, and extreme groups had significantly higher BPI pain interference and severity scores, PHQ-9 scores, and CSS-related disease numbers based on ANCOVA. Greater CSI-B numbers resulted in higher CSI-A scores (*p* < 0.001) and a higher odds ratio (*p* for trend <0.001). CSS-related symptoms were associated with pain severity, pain interference with daily activities, and depressive symptoms in various pain-related diseases.

**Conclusions:**

Our findings suggest that CSS may participate in these conditions as common pathophysiology.

## 1. Introduction

Central sensitization represents enhanced functions of circuits in nociceptive pathways and an abnormal state of the nociceptive systems, resulting from the remarkable plasticity of the somatosensory nervous system [1]. Due to alterations in the somatosensory system from high- to low-threshold pain hypersensitivity in this condition, central sensitization pain occurs in the absence of noxious stimuli. Central sensitization is characterized by allodynia in which pain is induced by nonnoxious stimuli [2], hyperalgesia [3, 4], and widespread pain [5], which was described initially only in animal models [6, 7]. Central sensitization is defined as increased responsiveness of nociceptive neurons in the central nervous system to their normal or subthreshold afferent input according to the International Association for the Study of Pain [8]. It should be noted that because direct electrophysiological recordings from central nervous systems are not performed in humans, human sensory profiling relies on proxies, which are believed to reflect central sensitization.

Central sensitization plays a role in fibromyalgia, a refractory pain disease in which alteration of central nociceptive processing occurs and pain can be worsened by psychological factors [9]. In patients with migraine, impaired descending pain inhibitory control assessed by conditioned pain modulation has been reported in an experimental pain setting compared with healthy controls [10], and central sensitization may contribute to acute allodynia and headache chronification [11]. In patients with restless legs syndrome, which is characterized by an abnormal, often painful sensation in the legs, levodopa-responsive hyperalgesia is thought to be mediated by central sensitization to A beta-fibers [12]. Patients with irritable bowel syndrome, a common gastrointestinal disorder, show extraintestinal symptoms suggestive of a central hyperalgesic state and have cutaneous hyperalgesia observed in other chronic pain diseases, which is abnormal processing of central nociception [13]. Chang and Lu suggested that irritable bowel syndrome and migraine share many similarities in associated comorbidities and possible pathogenesis regarding pain characteristics and treatment, indicating that they could be the same spectrum disorder involving central sensitization [14]. Central sensitization may be related to a relationship among migraine, inflammatory diseases, and psychiatric disorders [15]; additionally, a relationship among epilepsy, migraine, and psychiatric disorders [16] and a possible role of central sensitization on their pathophysiology have been discussed. A systematic review on central sensitization in chronic whiplash showed that hypersensitivity of the central nervous system plays an important role in persisting pain complaints in patients with chronic whiplash, but the underlying mechanisms are unclear [17].

Based on these findings, central sensitization plays a key role in sustained and amplified pain observed in certain proportions of refractory pain-related diseases, and it can participate in the pathophysiology of pain chronification, contributing to pain-related disability and inducing comorbid psychiatric symptoms and nonspecific symptoms such as fatigue and dizziness. These consequences not only negatively impact daily activity and the quality of life of affected individuals but also reduce social productivity. However, the role of central sensitization in refractory pain-related diseases is not yet fully understood. Investigating the effect of central sensitization in various diseases requires a large sample of pain-related diseases with a multicenter collaboration.

The Central Sensitization Inventory (CSI) is a self-administered questionnaire addressing symptoms associated with central sensitization and screening for several diseases related to central sensitization, including chronic headaches, restless legs syndrome, fibromyalgia, chronic fatigue syndrome, and multiple chemical sensitivity. CSI has been widely used and validated for the assessment of symptoms related to central sensitization syndrome (CSS) [18]. In this study, we performed a multicenter study using the Japanese version of the CSI [19] to assess symptoms related to central sensitization in patients with neurological, psychological, and pain disorders.

## 2. Methods

### 2.1. Participants

A total of 567 outpatients (199 M/368 F; age, 57.6 ± 18.1 years) were recruited from neurological psychiatric pain clinics in a multicenter setting between March 2018 and April 2019. Patient diagnoses in this multicenter study included migraine, tension-type headache, medication overuse headache, Parkinson's disease, stroke, restless legs syndrome, neuropathy, fibromyalgia, complex regional pain syndrome, cervical/lumbar spine disease, chronic fatigue syndrome, and depression. After excluding missing data on CSI, 551 outpatients (193 M/358 F; age 57.1 ± 18.0 years) were finally included. Additionally, 6,135 residents as general healthy controls were recruited from Utsunomiya city located adjacent to Dokkyo Medical University Hospital. When they had undergone an annual health check-up, they answered the questionnaire. Excluding missing values for sex, age, and CSI, 5,188 (2,036 M/3,152 F; age 64.7 ± 12.1 years) were included in this study.

The present study was carried out in accordance with the Declaration of Helsinki and was approved by the institutional review boards of Dokkyo Medical University Hospital and other participating facilities. All participants provided written informed consent to participate in the study.

### 2.2. Clinical Assessments

Participants completed questionnaires including smoking, caffeine intake, alcohol consumption, and body mass index. CSS-related symptoms were assessed by the Japanese version of the CSI, comprising parts A and B [19]. CSI-A scores 25 items for health-related and CSS-related somatic symptoms (score, 0–100), and CSI-B screens whether subjects have previously been diagnosed with 10 specific CSS diagnoses: restless legs syndrome, chronic fatigue syndrome, fibromyalgia, temporomandibular joint disorder, migraine or tension headaches, irritable bowel syndrome, and multiple chemical sensitivity. Cronbach's *α* coefficients of CSI-A scores were 0.902 in the patient group and 0.894 in the control group in the present study. Patients were categorized into 5 groups based on CSI-A scores: subclinical, 0–29; mild, 30–39; moderate, 40–49; severe, 50–59; and extreme, 60–100 [19, 20]. A CSI-A score of ≥40 is reportedly the optimal cutoff for distinguishing subjects with CSS and those without [19]. Patients completed the Brief Pain Inventory (BPI) Japanese version, consisting of a pain severity score (means of items 3–6), assessing pain severity, and a pain interference score (mean of items 9A-9G), addressing pain interference with daily activities [21]. The Japanese version of the Patient Health Questionnaire (PHQ)-9, assessing depressive symptoms (scores, 0–27), was also completed [22].

### 2.3. Statistical Analysis

Patient and control groups were compared using the chi-square test or Fisher's exact test for categorical variables and Student's *t*-test or Mann–Whitney *U* test for continuous variables where appropriate. To compare the CSI-A score and CSI prevalence (CSI-A ≥ 40 points) between patients and control groups, a matching dataset was obtained using caliper matching with a standard deviation of 0.25 times the propensity score calculated by age, gender, smoking, alcohol, and caffeine intake. The effect sizes between the patient and control groups were calculated by formula (1) (*r* ≥ 0.1: small, ≥0.3: medium, ≥0.5: large) for the Mann–Whitney *U* test or formula (2) (*φ* ≥ 0.1: small, ≥0.3: medium, ≥0.5: large) for the chi-square test as follows formulas [23]:

(1) $$\begin{array}{c} \text{effect sizes}\left(\gamma \right)=\frac{Z}{\sqrt{n}}, \end{array}$$

where *Z* is the standard test statics from the Mann–Whitney *U* test and *n* is the sample size.

(2) $$\begin{array}{c} \text{effect sizes}\left(\varphi \right)=\sqrt{\frac{x^{2}}{n\left(M-1\right)}}, \end{array}$$

where *x*^2^ is chi-square value, *n* is sample size, *M* is the value with the fewest rows and columns.

For the patient group, one-way analysis of variance or the Kruskal–Wallis test was used for the analysis of continuous variables among groups where appropriate, and the chi-square test was used for the analysis of categorical variables. Post hoc tests with residual analysis and Benjamini–Hochberg test for a chi-square test and Bonferroni test for ANOVA or Kruskal–Wallis test were performed. The PHQ-9 score, BPI pain interference score, BPI pain severity score, and the number of CSS-related diseases (based on CSI-B) among the 5 groups classified by CSI-A scores were analyzed using analysis of covariance (ANCOVA) after adjustment for potential confounding factors followed by the post hoc Bonferroni test. The relationship between a number of CSS-related diseases (0–6) and CSI-A scores was also analyzed by ANCOVA followed by the Bonferroni test and multivariable logistic regression model after adjustment for potential confounding factors. Each missing value was excluded in every analysis. Two-tailed *p* values < 0.05 were considered statistically significant. IBM SPSS Statistics software version 24.0 (IBM SPSS, Inc., Tokyo, Japan) was used for statistical analyses.

## 3. Results

Table 1 shows the characteristics of the patient and control groups in this study. Among the patient groups, the mean CSI-A score was 28.1 ± 16.0, and 20.7% had a CSI-A score ≥ 40. Among the 10 CSS-related diseases (CSI-B), migraine or tension headache was the most frequent (42.5%), followed by neck injury (11.6%), depression (11.1%), restless legs syndrome (7.8), and so on. Compared with the control group, the patient group had a significantly greater CSI-A score and a number of CSS-related diseases on CSI-B (*p* < 0.001). Medium effect sizes (*r* = 0.37) for CSI-A scores and large effect sizes (*r* = 0.64) for CSI-B disease numbers were found between patients and control groups. Next, we performed a subanalysis comparing 526 patients and 523 propensity score-matched controls. The results replicated significant differences in CSI-A scores and CSS-related diseases on CSI-B among the groups (Table 2). The effect sizes for the CSI-A score and CSI-B were 0.37 and 0.64, respectively (Table 2).

There was a significant difference in age, sex, number of CSS-related diseases on CSI-B, BPI pain interference score, BPI pain severity score, and PHQ-9 score among the 5 groups classified according to CSI-A scores (Table 3). Post hoc tests showed that the proportion of women was higher in the severe group than in the subclinical score group. The mean age was significantly higher in the subclinical score group than in the mild and severe score groups. The number of 10 CSS-related diseases, BPI pain interference and severity scores, and PHQ-9 score of the mild, moderate, severe, and extreme score groups were significantly higher than those of the subclinical score group (Table 3).

**Figure 1:**
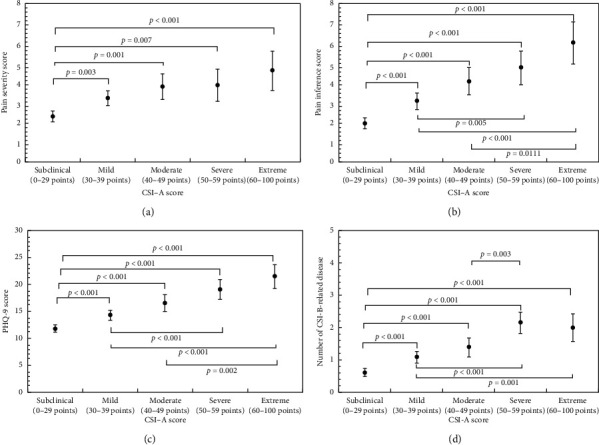
Relationship between clinical parameters and CSI-A score groups in the patients. Error bar indicates the 95% confidence interval. CSI: Central Sensitization Inventory; PHQ-9: Patient Health Questionnaire. using ANCOVA followed by post hoc comparison with the Bonferroni test. (a) and (b) were adjusted for sex, age, BMI, smoking, alcohol, caffeine and CSS-related diseases on CSI-B. (c) was adjusted for sex, age, BMI, smoking, alcohol, caffeine, CSS-related diseases on CSI-B, and BPI pain interference and severity score. (d) was adjusted for sex, age, BMI, smoking, alcohol, caffeine, PHQ-9 score, and BPI pain severity and interference score. (a) (*n* = 459), (b) (*n* = 474), (c) (*n* = 452), and (d) (*n* = 452).

ANCOVA followed by Bonferroni's test, after adjustments for confounding factors with sex, age, BMI, smoking, alcohol, and caffeine intake and the number of CSI-B or BPI or PHQ-9, showed that the CSI-A subclinical group had lower BPI pain interference and severity scores. Figures 1(a) and 1(b)), PHQ-9 (Figure 1(c)), and a smaller number of CSS-related diseases on CSI-B (Figure 1(d)) than the CSI-A mild, moderate, severe, and extreme groups. The PHQ-9 score, BPI pain interference and severity score, and a number of CSS-related diseases on CSI-B increased significantly with increasing CSI-A scores.

In Table 4, a greater number of CSI-B resulted in higher CSI-A scores (*p* < 0.001). Additionally, compared with the 0 CSS-related disease group on CSI-B, the 1, 2, 3, and 4–6 CSS-related disease groups showed significantly higher odds ratios (ORs). The *p* value for the trend test was <0.001 among 5 CSS-related disease groups classified according to the cutoff point (≥40) of CSI-A scores.

## 4. Discussion

In this study, we assessed symptoms related to CSS in patients with neurological, psychological, and pain disorders in a multicenter case-controlled setting. First, we showed that the CSI-A score and the number of CSS-related diseases on CSI-B were significantly greater in the patient group than in the control group. In addition, we calculated that the effect sizes for CSI-A and CSI-B between the patient and control groups were large. Next, we found that pain severity, the degree of pain interference with daily activities, and depressive symptoms worsened, and a number of CSS-related diagnoses increased as the CSI-A score increased across various pain-related conditions. Previously, the CSI-A score was related to pain intensity and pain interference in patients with musculoskeletal disorders [19]. Similar to our study results, the relationship of higher CSI-A severity with an increased comorbid CSS-related diagnosis and CSS-related symptoms, such as pain intensity, anxiety, depressive symptoms, disability, and sleep disturbances, were described in 763 patients with chronic spinal pain disorder [24]. In a study including 20 patients with musculoskeletal pain, meditation analysis found that the relationship among anxiety symptoms, depression symptoms, and pain intensity was totally mediated by central sensitization [25]. However, to the best of our knowledge, there have been no large sample studies of neuropsychiatric and pain disorders, including Parkinson's disease, migraine, and restless legs syndrome; psychological diseases, including depression; and various pain disorders with an appropriate control group, investigating CSS-related symptoms.

A large population-based study consisting of 8,930 respondents on a health survey showed that women reported poorer health scores than men did, particularly those aged between 30 and 40 years and over 70 years [26]. Therefore, to address the possible effect of age and sex on the CSI-A score and its impact, we used ANCOVA to adjust for age and sex to analyze all the relationships between CSI-A severity and clinical factors. In a study analyzing 1,160 sequelae of 289 diseases and injuries, among leading causes of years lived with disability, the main contributors to global years lived with disability were mental and behavioral disorders and musculoskeletal disorders. Low back pain, major depressive disorder, neck pain, other musculoskeletal disorders, anxiety disorders, and migraine, which were included in our study, were ranked within the top 10 causes [27]. Thus, in this questionnaire-based, multicenter survey, it was important to assess the impact of central sensitization in 551 Japanese patients with various pain-related diseases.

Our finding that, as the CSI-A score increased, pain-related disability and depressive symptoms worsened even after adjustment for CSS-related diseases on CSI-B suggests that this impact of central sensitization was not specific to certain diseases. Alternatively, central sensitization may relatively uniformly impact pain-related disability and psychiatric symptoms in various pain-related conditions. Klyne et al. [28] reported signs of central sensitization in acute low back pain recovery in many patients, but it may result in poor outcomes when combined with other psychological factors. Additionally, we suggest that central sensitization may play a role in the shared pathophysiology of these conditions. Among neurological diseases, in migraine, central sensitization may play a role in trigeminal nerve activation and cortical spreading depression [11]. The neuropeptide calcitonin gene-related peptide is abundantly found in the trigeminal ganglion, and its release from the peripheral terminals may participate in the enhancement of central sensitization [29]. Hyperexcitability of the central nervous system is a core mechanism of central sensitization. Reduced pain inhibition and activation of NDMA receptors in the central nervous system also contribute to persistent chronic pain, producing abnormal sensations such as allodynia and hyperalgesia [30].

We found a significant link between higher depressive symptoms and higher CSS burden (CSI-A scores) in this study. Brain limbic structures are innervated by serotoninergic median raphe neurons and noradrenergic neurons from the locus coeruleus, in which dysregulation of these neurons likely mediates features of psychiatric diseases such as depression and anxiety [31]. Thus, it is possible that altered, long-term central stimulation to brain regions, including the limbic system, can induce and enhance persistent central sensitization, contributing to chronic painful conditions in various pain-related diseases. Therefore, the management of central sensitization could be an important target for refractory pain-related diseases.

The limitations of this study include a cross-sectional design in patient analysis, and CSS-related diagnoses were made by participants with CSI-B and not by physicians. Relatively few participants had more than 3 CSS-related diseases; thus, the 95% CI of the OR among the 3 and 4–6 CSS-related disease groups was wide. However, because the trend test for the OR among the 5 CSS-related disease groups showed significant differences, a relationship between the number of CSS-related diseases and a CSI-A score of ≥40 was more likely. Regarding potential confounding factors for lifestyle, smoking, alcohol, and caffeine intake were the only options for multivariate analysis. Further prospective studies and more confounding factors are warranted to show a longitudinal impact of CSS on disease progression in a patient group. Finally, we used a validated questionnaire to assess CSS-related symptoms (CSI) [32]; however, CSI does not identify and measure central sensitization itself, and currently, there have been no validated tools to identify central sensitization. Additionally, its associations with central sensitization as assessed by experimental measures such as hyperalgesia, facilitated temporal summation, and impaired conditioned pain modulation have not yet been made [8].

## 5. Conclusion

We revealed that CSS-related symptoms were associated with pain severity, pain interference with daily activities, and depressive symptoms in various pain-related diseases in a multicenter setting after adjusting for confounders. Our findings suggest that CSS may participate in these conditions as common pathophysiology and may be a target for future treatment of these disorders.

## Figures and Tables

**Table 1:** Characteristics of the patient and healthy control group.

	Patient group			Control group			*p* value^a^
	*N*	(*n*) mean	(%) SD	*N*	(*n*) mean	(%) SD	
Sex, female, *n* (%)	551	358	65.0	5188	3152	60.8	0.053
Age, mean, SD (yr.)	551	57.1	18.0	5188	64.7	12.1	<0.001
BMI, mean, SD (kg/m^2^)	530	23.2	4.1		—		
Smoking, yes, *n* (%)	542	99	18.3	5035	325	6.5	<0.001
Alcohol intake, yes, *n* (%)	538	225	41.8	4731	1328	28.1	<0.001
Caffeine intake, yes, *n* (%)	543	501	92.3	4124	2835	68.7	<0.001
CSI-A score, mean, SD, points	551	28.1	16.0	5188	15.8	11.8	<0.001^b^
≥40 points, *n* (%)		114	20.7		242	4.7	<0.001
CSI-B, mean, SD, number of 10 diseases	551	0.99	1.14	5188	0.01	0.09	<0.001^b^
Migraine or tension headaches, yes, *n* (%)		234	42.5		5	0.1	<0.001
Neck injury (including whiplash), yes, *n* (%)		64	11.6		0	0.0	<0.001^b^
Depression, yes, *n* (%)		61	11.1		35	0.7	<0.001
Restless legs syndrome, yes, *n* (%)		43	7.8		0	0.0	<0.001^b^
Temporomandibular joint disorder, yes, *n* (%)		40	7.3		0	0.0	<0.001^b^
Anxiety or panic attacks, yes, *n* (%)		39	7.1		0	0.0	<0.001^b^
Irritable bowel syndrome, yes, *n* (%)		30	5.4		2	0.0004	<0.001^b^
Fibromyalgia, yes, *n* (%)		15	2.7		0	0.0	<0.001^b^
Chronic fatigue syndrome, yes, *n* (%)		14	2.5		0	0.0	<0.001^b^
Multiple chemical sensitivities, yes, *n* (%)		5	0.9		0	0.0	0.031^b^
BPI score, mean, SD, points							
Pain interference score	501	2.75	2.69		—		
Pain severity score	516	2.83	2.28		—		
PHQ score, mean, SD, points	550	13.7	6.1		—		

BMI: body mass index; CSI: Central Sensitization Inventory; BPI: Brief Pain Inventory; PHQ: Patient Health Questionnaire. ^a^Using a chi-square test or Student's *t*-test; ^b^using Fisher's exact test or a Mann–Whitney *U* test.

**Table 2:** Central Sensitization Inventory of the propensity score-matched patient and control groups.

	Patient group (*n* = 526)		Control group (*n* = 523)		*p* value^a^	Effect size^c^
	(*n*) mean	(%) SD	(*n*) mean	(%) SD		
Sex, female, *n* (%)	338	64.3	343	65.5	0.653	—
Age, mean, SD (yr.)	57.2	17.7	58.7	14.3	0.132	—
Smoking, yes, *n* (%)	97	18.4	108	20.7	0.367	—
Alcohol intake, yes, *n* (%)	221	42.0	206	39.4	0.387	—
Caffeine intake, yes, *n* (%)	488	92.3	493	94.3	0.328	—
CSI-A score, mean, SD, points	28.1	15.7	17.2	12.3	<0.001^b^	0.37
≥40 points, *n* (%)	105	20.0	29.0	5.5	<0.001	0.22
CSI-B, mean, SD, number of 10 diseases	1.01	1.15	0.01	0.123	<0.001^b^	0.64

The propensity score was calculated by age, sex, smoking, alcohol, and caffeine intake. CSI: Central Sensitization Inventory. ^a^Using a chi-square test or Student's *t*-test; ^b^using a Mann–Whitney test; ^c^effect size was calculated by *r* (≥0.1: small, ≥0.3: medium, and ≥0.5: large) for a Mann–Whitney *U* test or *φ* (≥0.1: small, ≥0.3: medium, and ≥0.5: large) for a chi-square test.

**Table 3:** Differences in each demographic and clinical parameter among the five CSI-A groups.

	*N* ^c^	Subclinical (i) (0–29)		Mild (ii) (30–39)		Moderate (iii) (40–49)		Severe (iv) (50–59)		Extreme (v) (60–100)		*p* value^a^	Post hoc test
Sex, female, *n* (%)	551	192	61.0	82	67.2	36	69.2	33	84.6	15	65.2	0.049	i vs. iv
Age, yr., *n* mean (SD)	551	315	59.8 (17.9)	122	53.7 (18.6)	52	53.6 (15.9)	39	50.8 (16.0)	23	57.3 (18.9)	0.001	i vs. ii, iv
BMI (kg/m^2^), *n* mean (SD)	530	303	22.9 (4.0)	117	23.3 (3.7)	49	23.8 (4.7)	38	23.9 (4.0)	23	24.1 (5.6)	0.269	—
Smoking, *n* (%)	542	49	15.7	24	20.2	14	27.5	8	22.2	4	17.4	0.288	—
Alcohol intake, *n* (%)	538	131	42.4	56	46.7	20	41.7	12	30.9	5	22.7	0.168	—
Caffeine intake, *n* (%)	543	283	91.6	114	93.4	49	96.1	37	94.5	18	81.8	0.266	—
CSS-related diseases on CSI-B, *n*, mean (SD)	551	315	0.6 (0.7)	122	1.1 (1.0)	52	1.4 (1.3)	39	2.1 (1.5)	23	2.0 (1.4)	<0.001^b^	i vs. ii, iii, iv, v ii vs. iv
Restless legs syndrome		15	4.8	12	9.8	5	9.6	8	20.5	3	13.0	0.006	i vs. iv
Chronic fatigue syndrome		1	0.3	2	1.6	2	3.8	8	20.5	1	4.3	<0.001	i vs. iv
Fibromyalgia		0	0.0	3	2.5	3	5.8	8	20.5	1	4.3	<0.001	i vs. iv
Temporomandibular joint disorder		9	2.9	9	7.4	6	11.5	11	28.2	5	21.7	<0.001	i vs. iii, iv, v
Migraine or tension headaches		112	35.6	67	54.9	26	50.0	19	48.7	10	43.5	0.003	i vs. ii
Irritable bowel syndrome		7	2.2	6	4.9	5	9.6	7	17.9	5	21.7	<0.001	i vs. iv, v
Multiple chemical sensitivities		3	1.0	1	0.8	1	1.9	0	0.0	0	0.0	0.881	—
Neck injury (including whiplash)		21	6.7	13	10.7	12	23.1	9	23.1	9	39.1	<0.001	i vs. iii, iv, v
Anxiety or panic attacks		7	2.2	8	6.6	9	17.3	10	25.6	5	21.7	<0.001	i vs. iii, iv, v
Depression		13	4.1	19	15.6	10	19.2	11	28.2	8	34.8	<0.001	i vs. iii, iv, v
BPI: pain interference score, *n*, mean (SD)	501	281	1.8 (2.2)	113	3.0 (2.4)	47	4.1 (2.4)	37	4.9 (2.9)	23	7.2 (2.4)	<0.001^b^	i vs. ii, iii, iv, v
													ii, iii, vs. v
BPI: pain severity score, *n*, mean (SD)	516	291	2.2 (2.1)	118	3.2 (2.2)	47	3.9 (2)	37	4.0 (2.1)	23	5.1 (2.0)	<0.001^b^	i vs. ii, iii, iv, v
													Ii vs. v
PHQ-9 score, *n*, mean (SD)	550	314	10.9 (3.5)	122	14.8 (5.4)	52	17.5 (5.7)	39	20.8 (6.3)	23	24.7 (8.3)	<0.001	i vs. ii, iii, iv, v
													ii vs. iii, iv, v
													iii vs. v

BMI: body mass index; CSI: Central Sensitization Inventory; BPI: Brief Pain Inventory; PHQ: Patient Health Questionnaire. ^a^Using a chi-square test or one-way analysis of variance (ANOVA) and post hoc test with residual analysis and Benjamini–Hochberg test for a chi-square test and Bonferroni test for ANOVA were performed. ^b^Using a Kruskal–Wallis test, a post hoc test with the Bonferroni test was performed. ^c^Missing values were excluded

**Table 4:** Relationship between the number of CSS-related diseases on CSI-B and CSI-A scores.

CSS-related diseases on CSI-B	*n*	%	CSI-A score				CSI-A score (more than 40 points)			
			Mean^b^	SE	*p* value^a^	Post hoc test^c^	OR^d^	95% CI	*p* value	*p* for trend
0 diseases	178	39.4	23.9	0.8	<0.001	0 vs. 1, 2, 3, 4–6	Ref.			<0.001
1 disease	171	37.8	29.5	0.8		1 vs. 2, 3, 4–6	2.70	1.15, 6.34	0.023	
2 diseases	63	13.9	34.7	1.3		2 vs. 4–6	4.86	1.78, 13.25	0.002	
3 diseases	25	5.5	36.3	2.1			20.09	5.16, 78.20	<0.001	
4–6 diseases	15	3.3	43.2	2.7			38.59	6.97, 213.58	<0.001	

CSS = central sensitization syndrome; CSI = Central Sensitization Inventory. ^a^Using a general linear model with ANCOVA; ^b^after adjustment for sex, age, BMI, smoking, alcohol, caffeine, BPI pain severity score, BPI pain interference score, and PHQ-9 score; ^c^using a Bonferroni test (*p* < 0.05); ^d^using a multivariable logistic model after adjusting for sex, age, BMI, smoking, alcohol, caffeine, BPI pain severity score, BPI pain interference score, and PHQ-9 score.

## Data Availability

The datasets from this study are available from the corresponding author upon reasonable request.
